# Detection of avascular necrosis on routine diffusion-weighted whole body MRI in patients with multiple myeloma

**DOI:** 10.1259/bjr.20180822

**Published:** 2019-02-09

**Authors:** Naeem Ahmed, Priya Sriskandarajah, Christian Burd, Angela Riddell, Kevin Boyd, Martin Kaiser, Christina Messiou

**Affiliations:** 1 Department of Radiology, The Royal Marsden Hospital, London, UK; 2 Department of Molecular Pathology, Institute of Cancer Research, Sutton, UK; 3 Department of Haematology, The Royal Marsden Hospital, Sutton, UK; 4 Department of Radiology, The Royal Marsden Hospital and Faculty of Radiotherapy and Imaging Institute of Cancer Research, London, UK

## Abstract

**Objective:**

Current therapies for multiple myeloma, which include corticosteroids, increase risk of avascular necrosis. The aim of this study was to assess incidental detection of femoral head avascular necrosis on routine whole body MRI including diffusion weighted MRI.

**Methods:**

All whole body MRI studies, performed on patients with known multiple myeloma between 1 January 2010 to 1 May 2017 were assessed for features of avascular necrosis.

**Results:**

650 whole body MR scans were analysed. 15 patients (6.6%) had typical MR features of avascular necrosis: 2/15 (13.3%) had femoral head collapse, 4/15 (26.7%) had bilateral avascular necrosis and 9/15 (60%) were asymptomatic.

**Conclusion:**

This is the first report of avascular necrosis detected on routine whole body MRI in patients with multiple myeloma. Targeted review of femoral heads in multiple myeloma patients undergoing whole body MR is recommended, including in patients without symptoms.

**Advances in knowledge:**

Whole body MR which includes diffusion-weighted MRI is extremely sensitive for evaluation of bone marrow. Although whole body MRI is primarily used for evaluation of multiple myeloma disease burden, it also presents an unique opportunity to evaluate the femoral heads for signs of avascular necrosis which can predate symptoms.

## Introduction

Multiple myeloma (MM) is a malignant plasma cell disease which predominately affects the axial skeleton and proximal long bones.^1^ The National Institute for Health and Care Excellence (NICE) in England currently recommends that imaging is offered to all patients suspected of myeloma, and to consider whole body MRI as first-line imaging for detection of myeloma bone involvement.^2^ Whole body MRI has also been recommended in patients with myeloma with serological relapse or disease progression^2^ and at the Royal Marsden Hospital is also used for assessing response in high risk groups. Recent consensus from the International Myeloma Working Group is also influencing increased used of whole body MRI.^3^ Whole body diffusion-weighted MRI has become a core component of whole body MRI protocols in oncology due to its high sensitivity, relative speed and quantitative capabilities.^4^

Patients on treatment for multiple myeloma are at risk of avascular necrosis (AVN). Current first-line antineoplastic and first relapse treatment recommendations include the addition of corticosteroids, usually dexamethasone, alongside high-dose chemotherapeutic agents.^2^ Corticosteroids have been shown to reduce blood flow to the affected bone, and AVN is thought to be sequelae of impeded blood supply causing death of osteocytes.^5^ Further risk factors include diabetes, cumulative dexamethasone dose, radiotherapy, alcoholism, infections, coagulopathies and autoimmune conditions.^6^

Early detection of AVN may allow for treatment regimen modification, including lowering of the cumulative dose of corticosteroids to minimise risk of femoral head collapse and regular monitoring of those at higher risk.^5^ Early stage AVN of the femoral heads is painless and becomes painful with disease progression. MRI is the most sensitive diagnostic imaging test for AVN with a reported sensitivity of 90–100% and specificity of 100%.^7,8^

The aim of this study was to assess the incidence of AVN detection on routine whole-body MRI including diffusion-weighted MR, in patients with multiple myeloma.

## Methods and materials

Local institutional review board approval was obtained. We performed a retrospective analysis of all whole body MRI studies performd on patients with an established diagnosis of MM between 1 January 2010 and 1 May 2017 at the Royal Marsden Hospital. Patients with incomplete scan protocols were excluded.

### Scan protocol

Using an Avanto and Aera 1.5 T system (Siemens, Erlangen, Germany) a WB study was achieved by the serial acquisition of contiguous body regions. All subjects were scanned supine with arms by their sides. Coil elements were positioned from skull vertex to knees. Sagittal *T*_1_ weighted images [repetition time (TR) 590 ms, echo time (TE) 11 ms, field of view (FOV) 400 mm, slice thickness 4 mm], and *T*_2_ weighted images (TR 2690 ms, TE 93 ms, FOV 400 mm, slice thickness 4 mm) of the spine were acquired, followed by axial diffusion-weighted sequences (single-shot double spin echo echoplanar technique with short tau inversion-recovery fat suppression in free breathing) using b-values of 50 and 900 s/mm^2^ applied in three orthogonal directions and combined to the isotropic trace images. Diffusion-weighted images were acquired in multiple contiguous stations of 50 slices per station (slice thickness 5 mm, no gap, FOV 430 mm, phase direction anteroposterior, parallel imaging (GRAPPA) factor 2, TR 14800 ms, TE 66 ms, inversion time (TI) 180 ms, voxel size 2.9 x 2.9 x 5 mm, number of signal averages 4, matrix 150 × 150, bandwidth 1960 Hz per pixel). Axial *T*_1 _weighted Vibe Dixon three-dimensional gradient echo breath-hold sequences (52 slices per slab, FOV 470 mm, TR/TE 7/2.38, 4.76 ms, flip angle 30, matrix 192 × 192) were also acquired, matching the acquisition stacks and partition thickness to the DWI.

### Data collection

All scans were initially reported by a consultant radiologist with a specialist interest in MM for whom review for features of AVN is standard practice. All whole body MRI studies were retrospectively independently reviewed to assess for the presence of features of AVN and signs of progressing AVN. Features of femoral head AVN were defined as: low serpiginous signal band on axial *T*_1_ weighted Vibe Dixon and femoral head fragmentation/collapse assessed on all sequences. Oedema was also evaluated on b50 DW MRI and ADC maps. Clinical history including dexamethasone, radiotherapy and bisphosphonate use and disease activity as per International Myeloma Working Group criteria^3^ were recorded from the Electronic Patient Records.

## Results

**Figure 1.**
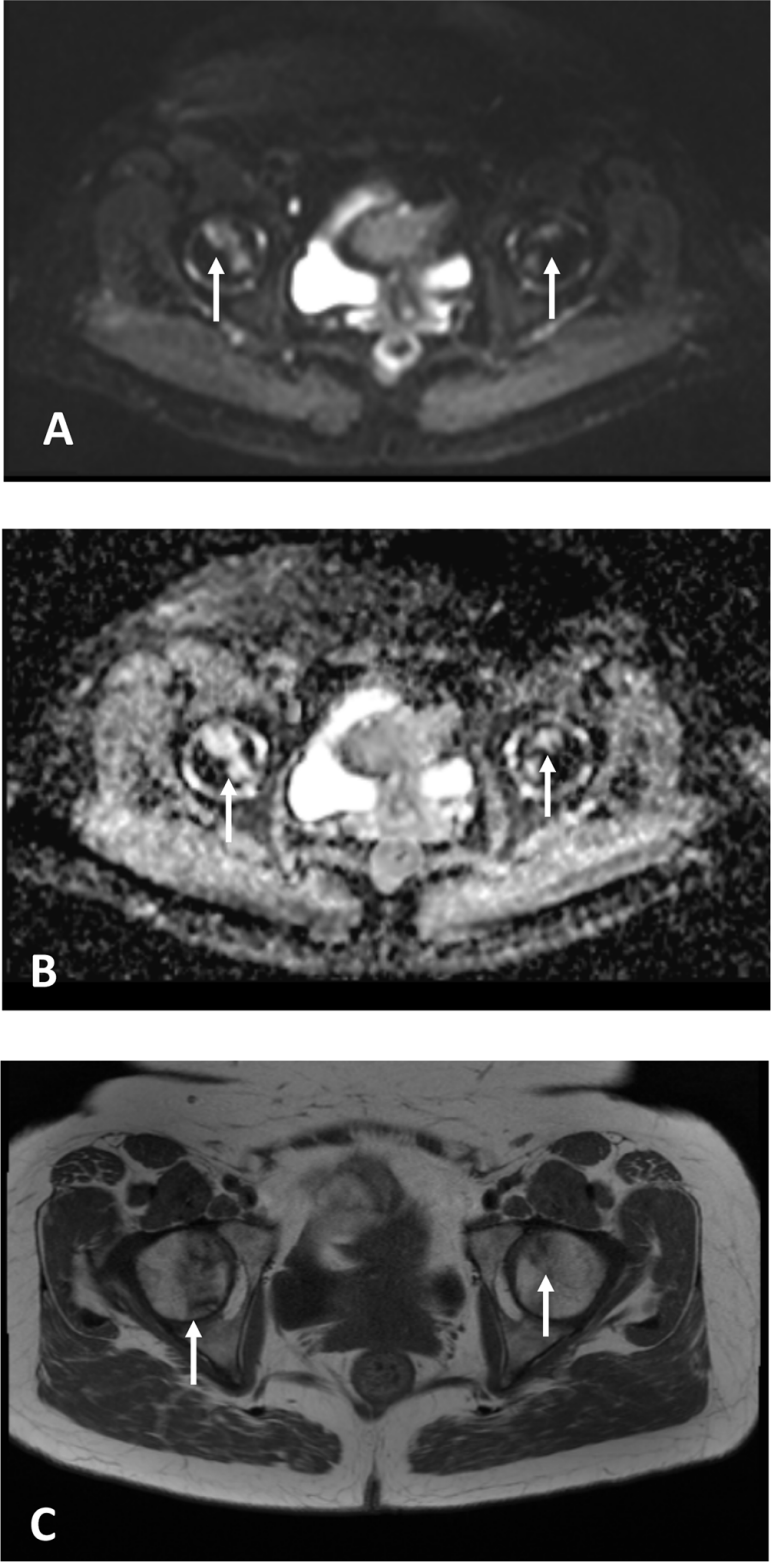
Axial MRI images (A: b50 diffusion-weighted MRI, B: corresponding apparent diffusion coefficient map, C: *T*_1_W MRI) of a 52-year-old lady on thalidomide and dexamethasone maintenance therapy. Bilateral subchondral signal changes (arrows) are evident on the diffusion weighted MRI (A, B) and on *T*_1_W MRI (C). No femoral symptoms were reported at the Mme of imaging which was performed for back pain. LeP hip replacement was performed 2 years following on from this scan due to pain.

Between 1 January 2010 and 1 May 2017, 650 whole body MR scans were performed in 226 patients with an established diagnosis of MM. 15 of these patients (6.6%) (10 male, 5 female, median age 66.5 years (range 31–74) were identified to have typical MR features of AVN.4/15 (27%) had a known history of AVN. Indications for scan included disease assessment post-treatment (6/15), biochemical progression (5/15) or reported bone pains at other sites (1/15). In addition to subchondral serpiginous lines, and oedema which were evident on all sequences (Figure 1), in each of the 15 patients, 2/15 had femoral head collapse and 4/15 had bilateral AVN. 10/15 patients had active disease according to IMWG criteria and 8/15 were being treated with steroid-combination regimens. Median cumulative dexamethasone dose received by these patients was 540 mg (range 80–5040 mg) while 3/15 had received prior pelvic radiotherapy. None of these patients had a background of diabetes. All patients were receiving bisphosphonates on a monthly to bi-monthly basis. 6/15 patients reported symptoms in the affected hip(s) at the time of AVN detection. 10/15 were referred for orthopaedic review.

The majority of these patients continued to have follow-up scans (9/15) for disease assessment, with a mean duration of 7.2 months (range 2–12).

During surveillance, 8/9 patients showed features of worsening AVN on follow-up MRI scans, including worsening femoral head oedema and increasing effusions. Of these, 4/8 reported worsening symptoms in the affected hip(s) and subsequently required surgical intervention with total hip replacements. All other patients were managed conservatively and are continuing to be reviewed by the orthopaedic team.

## Discussion

AVN is a known complication of steroid therapy and therefore patients with MM are at risk. Symptoms often occur at late stage femoral head collapse at which time non-surgical treatment options are limited. In keeping with previous published data, which has shown a 9% incidence of AVN in patients^5^ with MM, the incidence of AVN in our MM patients imaged with routine whole body MRI was low (6.6%). However, we have demonstrated that asymptomatic AVN can be detected before femoral head collapse which provides a window of opportunity for minimally invasive management. Although abnormal signal on diffusion-weighted MRI in the femoral heads has been reported in children with AVN this is the first report of diffusion-weighted MRI for detection of AVN in adults and the first report of asymptomatic femoral head AVN detection on whole body MRI in patients with MM.^9^ The retrospective nature of this data collection, limited number of cases and lack of standardised follow-up poses limitations and we propose prospective studies to further establish the natural history and risk factors for MM patients developing AVN. Studies on the relative contributions of the different imaging sequences are also desirable.

AVN can have a substantial effect on a patient’s morbidity and quality of life as AVN progresses to femoral head collapse and osteoarthritis.^10^ Femoral head AVN is a rapidly progressing pathology reportedly with a 70–80% chance of femoral head collapse after 3 years.^10^ A further study examining hips in 50 patients with early MRI features of AVN, over a period of 49 months, reported 32% of the femoral heads had collapsed by 32 months.^11^

Whole body MR is useful for early detection of AVN in those that are asymptomatic and for monitoring progression. Despite having radiological features of AVN, 60% were asymptomatic and of those patients followed up 89% demonstrated more advanced imaging features of AVN.

MRI is the most propitious imaging modality in the investigation of early femoral osteonecrosis and asymptomatic contralateral disease. A combination of clinical and radiological findings guide orthopaedic surgeons in managing femoral head osteonecrosis. Early detection allows for prompt referral and consideration of non-surgical options.

Imaging drives management decision-making in femoral osteonecrosis. There are a number of imaging-based classification systems used to stratify femoral head osteonecrosis, *e.g.* the Ficat and Arlet classification.^12^ Measurement of the size of any cortical defect present, at the mid-sagittal and mid-coronal MR planes may be useful for decision-making and have been implicated in predicting future femoral head collapse.^13^ However, accurate measurements may require dedicated small FOV images but the need for such measurements will be guided by preference of the local orthopaedic surgeons.

Pharmacological therapy such as Alendronate (a bisphosphonate) which is routinely part of the myeloma regimen has been shown to prevent early femoral head collapse.^14^ However, despite all of our patients with MRI features of AVN were being treated with bisphosphonate agents.

Surgical options for early stage AVN are not yet standard practice. These aim to preserve the femoral head, include core decompression, either with or without bone grafting which involves drilling of small holes through the subchondral necrosis to stimulate a healing response through angiogenesis and reduce intraosseous pressure.^15^ The success of novel therapies such as bone tissue engineering which may offer reparative potential avoiding complications of surgery, will be reliant on early detection.^16^ For advanced femoral head osteonecrosis, with resulting subchondral collapse, treatment options are usually restricted to more extensive surgery such as total hip replacements and total hip resurfacing.

It is interesting to note that although the frequency of incidental findings is high in patients with myeloma undergoing whole body MRI (70 in 175 examinations).^17^ Wale et al reported that only 3% of whole body MRI scans in patients with myeloma resulted in the need for further investigation. Most incidental findings were not significant, however, as AVN can be a significant cause of morbidity for patients with myeloma routine review of the femoral heads on whole body MRI is recommended.

## Conclusion

Whole body MR will be increasingly used for the evaluation of patients with MM. Patients with MM are often on high dose steroid regimens and are at increased risk of femoral head osteonecrosis. Femoral head AVN can progress quickly and may have devastating consequences for the patient’s quality of life necessitating surgical intervention which may be challenging in the presence of co-morbidities.

This is the first study demonstrating that AVN can be detected on routine whole body MRI, including diffusion-weighted MRI in patients with MM. Evaluation of femoral heads in all multiple myeloma patients undergoing whole body MR should therefore be an integral part of the radiologist’s review.
